# Multiple Biliary Papillomatosis

**DOI:** 10.1155/1992/15823

**Published:** 1992

**Authors:** Mustafa Tireli, Adam Uslu

**Affiliations:** The Surgical Clinic of SSK Tepecik Hospital Izmir Turkey

## Abstract

Diffuse biliary papillomatosis is a rare bile duct tumour. We report a case of multiple biliary papillomatosis treated surgically with a transhepatic stent.

Diffuse biliary papillomatosis involving intra and extrahepatic bile ducts is extremely rare. It is regarded as having low grade malignant potential. In this report a case of diffuse biliary papillomatosis with obstructive jaundice is presented.


*HPB Surgery*, 1992, Vol. 6, pp. 125–128
Reprints available directly from the publisher
Photocopying permitted by license only

© Harwood Academic Publishers GmbH
Printed in the United States of America

### CASE REPORT

### MULTIPLE BILIARY PAPILLOMATOSIS

MUSTAFA TIRELI and ADAM USLU
*The Surgical Clinic of SSK Tepecik Hospital, Izmir, Turkey*

*(Received 9 March 1992)*

Diffuse biliary papillomatosis is a rare bile duct tumour. We report a case of multiple biliary papillomatosis treated surgically with a transhepatic stent.

Diffuse biliary papillomatosis involving intra and extrahepatic bile ducts is extremely rare. It is regarded as having low grade malignant potential. In this report a case of diffuse biliary papillomatosis with obstructive jaundice is presented.

KEY WORDS: Diffuse biliary papillomatosis, biliary stent

### CASE REPORT

A 70 year-old woman presented in March 1990 with obstructive jaundice. She had undergone cholecystectomy five years ago for cholelithiasis. Ultrasound examination revealed dilation of intra and extrahepatic bile ducts. Laboratory data confirmed obstructive jaundice. In March 1990 the patient underwent exploratory laparotomy. The common bile duct was found to be three centimeters in diameter and both the common and intrahepatic bile duct were filled with fragile, spongy polypoid material. The extent of the lesion permitted neither resection nor bilioenteric by-pass. Biliary drainage with a transhepatic stent was achieved with an indwelling size 20 French silastic tube inserted into the common bile duct. The lower end of the tube extended into the duodenum. Postoperative hepatic stent Cholangiography revealed dilation of the common duct and filling defects due to papillomatosis (Figure 1). Microscopic examination of curettings of the common bile duct showed tubulo-villous adenoma (Figure 2), and epithelial dysplasia; cell atypia was not observed. We recommend daily saline flushes and routine changing of the silastic stent fluoroscopically over guidewires whenever it is irreversibly obstructed by papillomatosis. However, the patient was free of symptoms at follow-up in March 1992.

### DISCUSSION

Diffuse intra and extrahepatic biliary papillomatosis is an extremely rare tumour of

Address correspondence to: Dr Mustafa Tireli, SSK Tepecik Hospital, Izmir, Turkey

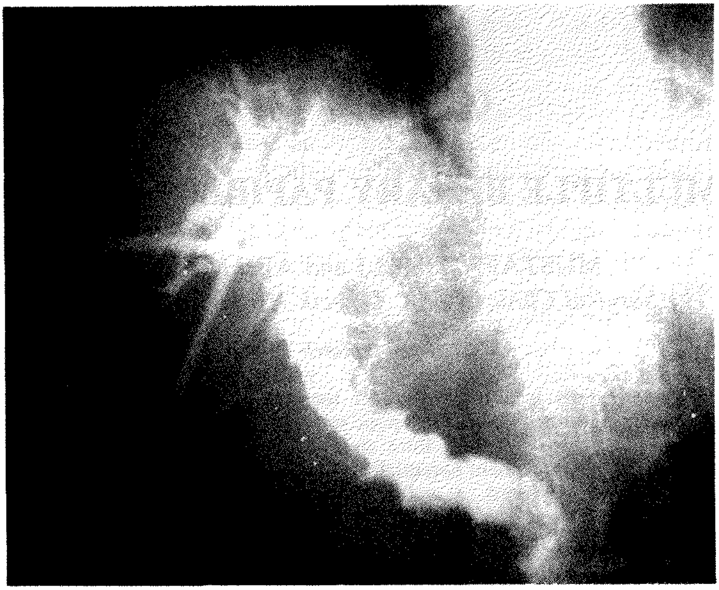

**Figure 1** Postoperative stent cholangiography demonstrated filling defects due to biliary papillomatosis.

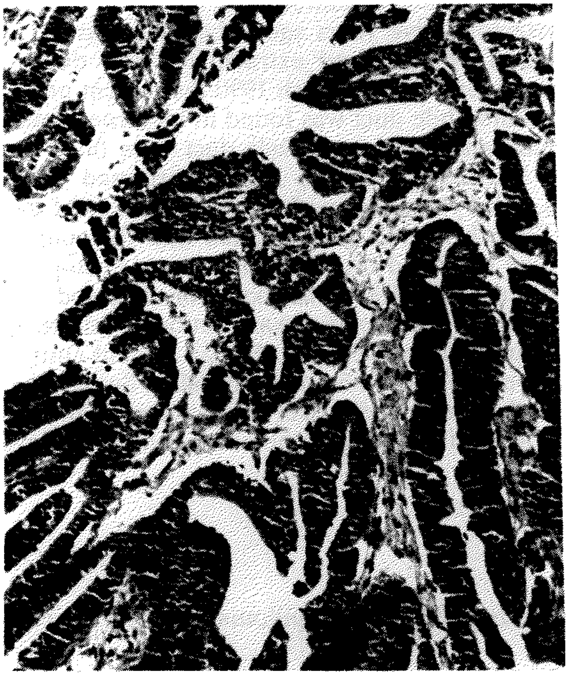

**Figure 2** Microscopic examination of curretting material of bile duct showed tubulo villous adenoma.

the bile duct. The number of recorded cases was nearly 30 up to now[1,2]. Some authors postulated that this lesion has frank malignant potential[2,4,5,6]. Gouma *et al.*[1], reported malignant changes in three of the 14 (21 per cent) reported cases.

This lesion progresses slowly and remains latent for long periods[4]. The most common clinical finding is obstructive jaundice[1,4,5,7], caused by the fragmentation of papillary projections[1,3]. Preoperative diagnosis may be difficult but current widespread availability of percutaneous transhepatic or endoscopic retrograde cholangiography has aided the diagnosis by identifying filling defects in bile ducts[2,4,5].

The treatment of biliary papillomatosis is surgery[1,3,4,7,8]. Surgical methods can be classified in two groups. The first method involves curative resection by means of hepatic lobectomy and resection of the common bile duct[5]. This method is performed when papillomatosis is confined to a single lobe and the extrahepatic bile ducts[1,3,5]. But recurrences have been observed in the remaining ducts after this form of treatment[3,5]. The overall survival exceeds five years in three of the five patients who had curative surgery[3]. The second method is palliative techniques consisting of biliary drainage. Bilioenteric anostomosis, curettage and T-tube or biliary stent drainage are the main techniques used[1,3,4,6,7]. The results of palliative methods are generally poor[3,7], and reoperation is performed for the relief of papillary reobstruction of the bile ducts in most cases[7,8]. Gouma *et al.*[1], reviewed 12 cases and reported 80 per cent local recurrence with intermittent obstructive jaundice and acute cholangitis. The mean survival time in this group was 28 months. Curative surgery is not applicable for the patients with diffuse involvement of intra and extrahepatic biliary tree, curettage and biliary drainage with transhepatic stent is the preferred palliative method in this group[1,3,5].

### *References*

1. Gouma, D.J., Mutum, S.S., Benjamin, I.S. and Blumgart, L.H. (1984) Intrahepatic biliary papillomatosis. *Br. J. Surg.*, **71**, 72
2. Bottger, T., Sorger, K., Jenny, E. and Junginger, T. (1989) Progressive papillomatosis of the intrahepatic and extrahepatic bile ducts. Case report. *Acta Chir. Scand.*, **155**, 125
3. Blumgart, L.H. (1985) Tumours of the gallbladder and bile duct, in: Schwarts S.I. and Ellis, H: Maingot's *Abdominal Operations*, Eighth Edition, Appleton Century Crofts, Norwalk, pp. 2015–2044
4. Neuman, R.D., Li Volsi, V.A., Rosenthal, N.S. *et al.* (1976) Adenocarcinoma in biliary papillomatosis. *Gastroenterology*, **70**, 779
5. Madden, J.J. and Smith, W.G. (1974) Multiple biliary papillomatosis. *Cancer*, **34**, 1316
6. Gertsch, P., Thomas, P., Baer, H. *et al.* (1990) Multiple tumours of the biliary tract. *Am. J. Surg.*, **159**, 386
7. Cattell, R.B. and Braasch, J.W. (1962) Polypoid epithelial tumours of the bile duct. *N. Eng. J. Med.*, **266**, 57
8. Eiss, S., Dominick, M. and Caedo, J.P. (1960) Multiple papillomas of the entire biliary tract. *Ann. Surg.*, **152**, 320

*(Accepted by S. Bengmark 15 March 1992)*

### INVITED COMMENTARY

Biliary tract papillomatosis is exceedingly rare and exceedingly difficult to cure. It is most unusual to have this condition localized to one segment or one lobe of the liver in addition to its common duct and hepatic duct localization. Of interest is the failure of this condition to involve the gallbladder, the benign histologic appearance of the papillomas and its invariable recurrence following anything short of excising the affected areas.

Palliation is possible by curettage and placement of drainage tubes. Repeat treatments of this nature are also possible, but sooner or later the intrahepatic density of papillomas exceeds the potential for palliation.

It would be of interest to treat these tumors by intracavitary radiation. Such treatment has yet to be recorded in the literature.

John Braasch
Lahey Clinical Medical Center
41 Mall Road
Burlington, MA 01805
USA

